# 

**Published:** 1878-07

**Authors:** 


*CONTENTS.*

| | Page |
|---|---|
| Sunshine . . . . . . . | 517 |
| Premature Death . . . . | 520 |
| Success in Life . . . . . . | 520 |
| Responsibility of Writers . | 522 |
| Influence of Mothers . . | 522 |
| How to be a Man . . . . | 523 |
| Controlling Temper . . . | 523 |
| Our Daughters . . . . . . | 524 |
| Unskilled Labor . . . . | 525 |
| Mental Rest . . . . . . . | 527 |
| The First Wedding . . . | 530 |
| Marriage . . . . . . . . . | 530 |
| A Valuable Table . . . . | 531 |
| The Mother . . . . . . . | 532 |
| The Motherless . . . . . | 533 |
| Health Ruined . . . . . | 534 |
| Appetite . . . . . . . . | 536 |
| Decision of Character . . | 537 |
| Comfort of the Poor . . | 538 |
| Medical Progress . . . . | 539 |
| Be Systematic . . . . . | 540 |
| A Physician's Life . . . . | 541 |
| Health Food for the Multitude . . . . . . . . | 542 |

